# Contrast Transfer Function-Based Exit-Wave Reconstruction and Denoising of Atomic-Resolution Transmission Electron Microscopy Images of Graphene and Cu Single Atom Substitutions by Deep Learning Framework

**DOI:** 10.3390/nano10101977

**Published:** 2020-10-06

**Authors:** Jongyeong Lee, Yeongdong Lee, Jaemin Kim, Zonghoon Lee

**Affiliations:** 1 Center for Multidimensional Carbon Materials, Institute for Basic Science (IBS), Ulsan 44919, Korea; jong022@unist.ac.kr (J.L.); lyd1227@unist.ac.kr (Y.L.); jmkim17@unist.ac.kr (J.K.); 2 Department of Materials Science and Engineering, Ulsan National Institute of Science and Technology (UNIST), Ulsan 44919, Korea

**Keywords:** deep learning, exit-wave reconstruction, denoising, single atom substitution, graphene, atomic resolution transmission electron microscopy

## Abstract

The exit wave is the state of a uniform plane incident electron wave exiting immediately after passing through a specimen and before the atomic-resolution transmission electron microscopy (ARTEM) image is modified by the aberration of the optical system and the incoherence effect of the electron. Although exit-wave reconstruction has been developed to prevent the misinterpretation of ARTEM images, there have been limitations in the use of conventional exit-wave reconstruction in ARTEM studies of the structure and dynamics of two-dimensional materials. In this study, we propose a framework that consists of the convolutional dual-decoder autoencoder to reconstruct the exit wave and denoise ARTEM images. We calculated the contrast transfer function (CTF) for real ARTEM and assigned the output of each decoder to the CTF as the amplitude and phase of the exit wave. We present exit-wave reconstruction experiments with ARTEM images of monolayer graphene and compare the findings with those of a simulated exit wave. Cu single atom substitution in monolayer graphene was, for the first time, directly identified through exit-wave reconstruction experiments. Our exit-wave reconstruction experiments show that the performance of the denoising task is improved when compared to the Wiener filter in terms of the signal-to-noise ratio, peak signal-to-noise ratio, and structural similarity index map metrics.

## 1. Introduction

In recent years, researchers have explored many new materials with unique properties. Among them, due to their excellent intrinsic properties, two-dimensional (2D) materials have been considered one of the most attractive materials for future applications, including semiconductor, batteries, fuel cells, sensors, and information technology [1]. The physical and chemical properties of 2D materials are greatly influenced by the microstructure, such as crystallinity, domain size, and atomic defects. Therefore, to understand the properties of 2D materials and to further tailor the properties required for a specific application, information on the atomic structure and composition of 2D materials is essential [2]. In recent years, atomic-resolution transmission electron microscopy (ARTEM) in real-time has become a major tool for observing the structure and dynamics of 2D materials. For example, the structure of 2D iron [3], the dynamics of graphene self-recovery [4], the growth of hexagonal boron nitride defects [5], and Cu single atoms in monolayer graphene that appear for a few seconds were investigated by ARTEM with a sub-Angstrom resolution. However, raw data obtained from a charge-coupled device (CCD) can often be misinterpreted because of the modification by the incoherence effect of the electron beam and the aberration of the optical lens system. To avoid this misinterpretation, exit-wave reconstruction, which calculates the electron state excluding the modification, was developed [6,7,8].

Conventional exit-wave reconstruction is an iterative method [6]. It is not suitable for real-time analysis because analyzing the intrinsic structure of an object requires a through focal series of images from the same specimen with different defocus values. Since the positions of atoms change easily, it is difficult to obtain a through focal series of images of the same structure. In addition, increasingly dynamic studies involving the rapid movement of atoms have been performed using ARTEM. To overcome this problem, a direct exit-wave reconstruction method using a single defocused image was suggested [7]. This method shows that the exit wave can be reconstructed from a single defocused image by using the correlation of phase and amplitude information within the adjacent space of the object. However, this method is not applicable to ARTEM due to the complex optical system and the incoherence effect of the electron beam between the object and surrounding free space. Therefore, the structural analysis of 2D material by ARTEM in real time has relied on the image simulation method [3,4,5].

The image simulation method estimates the CCD detection results using the exit wave, which is constructed from the atomic structure expected by humans and the contrast transfer function (CTF), which describes the aberration of the optical system [9]. The result of the image simulation is compared with the experimental raw data. This is reverse engineering conducted by matching the expected and actual structures using an image that relies highly on human know-how.

In our study, we present a deep learning framework for exit-wave reconstruction and denoising using the convolutional dual-decoder autoencoder (CDDAE) for ARTEM studies of the structure and dynamics of 2D materials. We apply a fast Fourier transform (FFT)-based deconvolution to CDDAE by including an image simulation equation with the precalculated CTF for the actual optical system conditions with measured aberrations by Zemlin tableau method [10]. The output of each decoder from CDDAE is the amplitude and phase of the reconstructed exit wave. Furthermore, the output of the total framework is the result of the image simulation calculation, applying CTF to the reconstructed exit wave, which is equivalent to the denoised Input image.

We demonstrate this CDDAE exit-wave reconstruction and denoising framework through ARTEM images of an experimental graphene dataset acquired by aberration-corrected TEM, which was operated at 80 kV. As a verification process, the experimental result was compared to the conventional image simulation verification method [11,12,13,14,15]. We also exhibit a direct identification of Cu single atom substitution in monolayer graphene through the comparison of the reconstructed exit wave. In addition, we evaluate the denoising performance by the signal-to-noise ratio (SNR), peak-signal-to-noise ratio (PSNR), and structural similarity index map (SSIM) compared to the conventional Wiener filter [16].

## 2. Related Works

### 2.1. Direct Exit-Wave Reconstruction from a Single Defocused Image

The exit wave can be expressed as the sum of the waves penetrating the object and the waves scattered by the object. The following equation represents the exit wave:

(1) $$\psi \left(r\right)=\psi_{\mathrm{penetrate}}\left(r\right)+\psi_{\mathrm{scattered}}\left(r\right)$$

where *r* is a vector in the exit wave surface. Additionally, the direct reconstruction of the exit wave can be extracted by using the relationship between the inverse Fourier transform of the CCD data ${\left|\phi \left(u\right)\right|}^{2}$ and the exit wave function ψ(r) with defocus z and wavelength λ [7]:

(2) $${ℱ}^{-1}\left[{\left|\phi_{z}\left(u\right)\right|}^{2}\right]=\int^{\;}{\tilde{\psi }}^{*}\left(r^{\prime }\right)\tilde{\psi }\left(r^{\prime }+r\right)dr\equiv f_{\mathrm{auto}}\left(r\right)$$

where:

(3) $$\tilde{\psi }\left(r\right)=\frac{1}{\lambda z}\psi \left(r\right)\exp\left(\frac{i\pi r^{2}}{\lambda z}\right)$$

(4) r=λzu

According to Equation (2), the exit wave can be reconstructed from a single defocused CCD image by using the correlation of the phase and the amplitude information retrieved by the autocorrelation of the scattered wave area surrounding the penetrating wave. To get the autocorrelation, the single defocus image needs enough penetrating wave area ($X_{\mathrm{free}}$ × $X_{\mathrm{free}}$), which is four times larger than the penetrating wave area ($X_{\mathrm{obj}}$ × $X_{\mathrm{obj}}$). The following Equation (5) should be satisfied.

(5) $$X_{\mathrm{obj}}\leq \frac{1}{\sqrt{5}}X_{\mathrm{free}}$$

According to the definition of the covalent radius, at least one of two adjacent atoms does not satisfy the aforementioned Equation (5) for ARTEM images of 2D materials.

### 2.2. Image Simulation Verification Method

Image simulation can be performed by applying the contrast transfer function (CTF), which describes the modification of the optic system, into the exit wave (vide infra) [11,12,13,14]:

(6) $${\left|\phi \left(u\right)\right|}^{2}={\left|\psi \left(u\right)⊗h\left(u\right)\right|}^{2}\;={\left|FT^{-1}\left[\Psi \left(k\right)H\left(k\right)\right]\right|}^{2}$$

where H(k), the contrast transfer function, can be expressed as follows [9]:

(7) H(k)=A(k)E(k)exp[−iχ(k)]

(8) $$\chi \left(k\right)=\;\frac{\pi }{2}C_{s}\lambda^{3}k^{4}-\pi \Delta fk^{2}$$

where A(k) is the aperture function, which describes the cutoff of the TEM aperture. E(k) is the envelope function that contains various factors of the lens, affecting the resolution.

To apply the image simulation method described above in the study of 2D materials, it is necessary to conduct the experiment in the following order:

1. Prediction of the atomic structure from the raw data obtained from the CCD.
2. Conversion of the expected atomic structure into the exit wave by using the multislice method.
3. Conduction of the image simulation by using the exit wave and CTF (Equation (6)).
4. Verification of the atomic structure by comparing the result of the image simulation to the raw data.
5. Iteration of the structure modulation until the simulated results of the expected atomic structure become similar to the actual image up to the desired level.

However, the abovementioned verification method of the image simulation with inverse engineering shows a limitation, insofar as the exit wave cannot be directly obtained from the actual image.

### 2.3. FFT-Based Image Deconvolution

FFT-based image deconvolution is a method involving an iterative deconvolution algorithm that can preserve image details [17,18]. A common blurred image (*y*) can be expressed as the following equation:

(9) y=k⊗x+ η

where *k* is the blur kernel, x is a latent image, and η represents additive noise.

The x is estimated as a reconstructed image $\hat{x}$ by finding the linear filter g, which is assumed to minimize the error ϵ, as shown below:

(10) $$\epsilon =E{\left|x-\hat{x}\right|}^{2}$$

(11) $$\hat{x}=gy$$

In the conventional Wiener deconvolution method, the blurred image can be restored by solving the following equation:

(12) $$\hat{x}=\;{ℱ}^{-1}\left(\frac{{ℱ}^{*}\left(k\right)ℱ\left(y\right)}{{\left|ℱ\left(k\right)\right|}^{2}+n/s}\right)$$

where n and s indicate the expected power spectra of the noise and image, respectively.

Since then, iterative error minimization research has been conducted by adding various regularization terms.

In recent years, research on convolutional neural networks (CNNs) has been actively conducted.

### 2.4. Autoencoder

An autoencoder is one of the deep learning structures organized by artificial neural networks [19], encoder networks, and decoder networks, connected by a latent vector. Each network is a sequential combination of an activation function and various regularization methods and hyperparameters, which include a linear and nonlinear operation, trained by a backpropagation algorithm. In the case of a basic autoencoder, the loss is the L2 normalization of the input data and output data, and it is minimized by training hyperparameters with backpropagation. The loss is defined as:

(13) $$\mathrm{loss}=\|x-g{\left(f\left(x\right)\right)\|}^{2}$$

where *f* is the encoder and *g* is the decoder. The basic autoencoder is studies in fields such as denoising [20], nonlinear dimensionality reduction [21], super resolution [22], and anomaly detection [23].

We found similarities between ARTEM image simulation (Equation (6)) and FFT-based image deconvolution (Equation (12)). Therefore, in our study, we propose a CDDAE framework for exit-wave reconstruction by applying the ARTEM image simulation to the FFT-based image deconvolutional method and autoencoder.

## 3. Materials and Methods

### 3.1. CDDAE Framework

**Figure 1:**
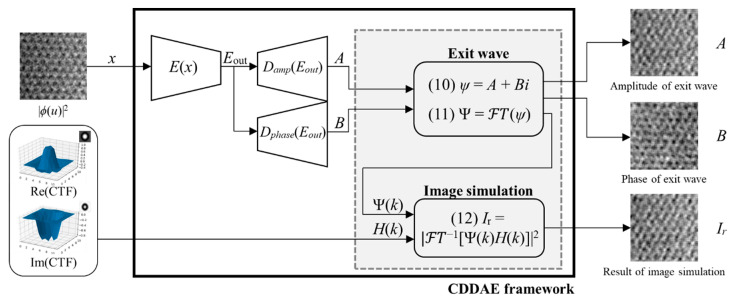
The structure of the convolutional dual-decoder autoencoder (CDDAE) framework. Input images are decoded to *A* and *B*, which correspond to the amplitude and phase of the exit wave. *I*_r_ is the result of the image simulation when applying the contrast transfer function (CTF) to the reconstructed exit wave.

Here, we propose a deep learning framework, CDDAE framework, which is composed of two main parts: an autoencoder part (*E, D*_amp_*, D*_phase_) and an image simulation part (Figure 1). The autoencoder part has an input image (*X*), which is an ARTEM image obtained from the CCD written in Equation (6) and described as ${\left|\phi \left(u\right)\right|}^{2}$ and which has the role of finding the relationship between *X* and the exit wave (ψ) from Equation (2) for the direct reconstruction of the exit wave. It is the variation of the convolutional autoencoder, composed of one encoder (*E*) and two decoders, an amplitude decoder (*D*_amp_) and a phase decoder (*D*_phase_). *E* consists of the first four sequential layers, and each layer has a rectified linear unit (ReLU) as an activation function. Then, *E*_out_, an output of *E*, is the latent space representation of the *X*. Suppose *A* is the output of *D*_amp_ with input *E*_out_, which decodes the amplitude of the exit wave from *E*_out_, and *B* is the output of *D*_phase_, which decodes the phase of the exit wave from *E*_out_. We set the activation function of each decoder to be a zero-centered hyperbolic tangent for the sensitive learning of each decoder to the sign of the wave. The end of each decoder has a convolutional layer, with a 1 × 1 size 1 kernel and no activation function, for merging results. Detailed information on the CDDAE network is provided in Table 1. 

The effective kernel size is 16, as the eight kernels with a size of 3 × 3 are sequentially stacked. In this case, the effective kernel size should be larger than the object size, because the convolution of the effective kernel and the object should be larger than the autocorrelation of the object (Equation (5)). The nonlinear property of the autoencoder reduces the effects of the factors unconsidered in the precalculated CTF, such as high order aberrations, attenuation factors, and noise. For the image simulation, we defined the exit wave *ψ*, which is a complex matrix, by using the outputs *A* and *B* of each decoder as follows:

(14) ψ=A+Bi

Furthermore, we could derive Ψ from the FFT of ψ as follows:

(15) Ψ=FT(ψ)

Suppose $I_{r}$ are the image simulation results obtained from assigning the precalculated CTF (Equation (7)) and *ψ* to Equation (6):

(16) $$I_{r}={\left|FT^{-1}\left[\Psi \left(k\right)H\left(k\right)\right]\right|}^{2}$$

CDDAE is a neural network that reconstructs the exit wave by reducing the loss function as follows:

(17) $$\mathrm{loss}=\|X-I_{r}\|{}^{2}$$

We use ADAM [24] to minimize the loss function, with 2e^−5^ as the initial learning rate and default parameters.

Learning the CDDAE framework by TEM images means discovering a nonlinear solution for reconstructing the exit wave and denoising an input image.

### 3.2. Training Data

The monolayer graphene sheet was synthesized by chemical vapor deposition (CVD) method [25,26], and ARTEM images were obtained from an aberration-corrected TEM (FEI, Hillsboro, OR, USA) with an acceleration voltage of 80 kV and a monochromator. The original 4000 ARTEM images have 1024 × 1024 px, and they were cropped into 128 × 128 px images, resulting in a 256,000 images dataset. One image contains approximately 300 carbon atoms. Since the same atom cannot be identified, about 76,800,000 objects were finally used to learn the CDDAE framework.

## 4. Results

### 4.1. Direct Exit-Wave Reconstruction from Single Image of Monolayer Graphene

To overcome incoherence effects and aberration problems, a numerical method can be applied by reconstructing the wave function at the exit surface of the specimen [6]. Exit-wave reconstruction aims to accurately interpret the atomic information by acquiring the exit wave prior to the influence of the CTF. To evaluate the CDDAE framework, it is first necessary to input the test set images into the network trained by training set images. Subsequently, the evaluation can be performed by comparing the resultant exit wave with that of the atomic structures obtained from the image simulation verification method.

**Figure 2:**
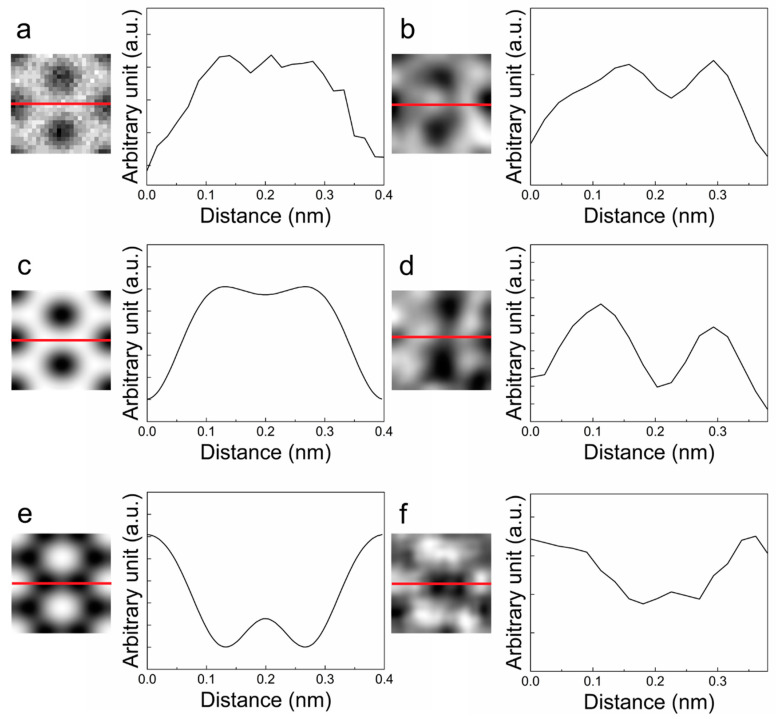
Images and intensity profiles of the simulated (**a,c,e**) and experimental (**b,d,f**) data by the CDDAE framework. The intensity profiles are plotted along the solid red lines from left to right in each image. (**a**) Image simulation result; (**b**) Input image; (**c**) Amplitude of simulated exit wave; (**d**) Amplitude of reconstructed exit wave; (**e**) Phase of simulated exit wave; (**f**) Phase of reconstructed exit wave.

The exit-wave simulation and image simulation of the monolayer graphene were conducted by MacTempas, version 2.4.9; Total Resolution: Berkeley, CA, USA, 2015. We verified the atomic structure by comparing it with the test set images. The carbon atoms appeared to be in white contrast from the simulated exit-wave amplitude, whereas they appeared to be in dark contrast in the case of the phase. The exit wave from the CDDAE framework also showed the same contrast. This result allowed us to conclude that the framework worked in obtaining the exit wave. Figure 2 shows the evaluated images.

### 4.2. Identification of Cu Single Atom from Single Image

**Figure 3:**
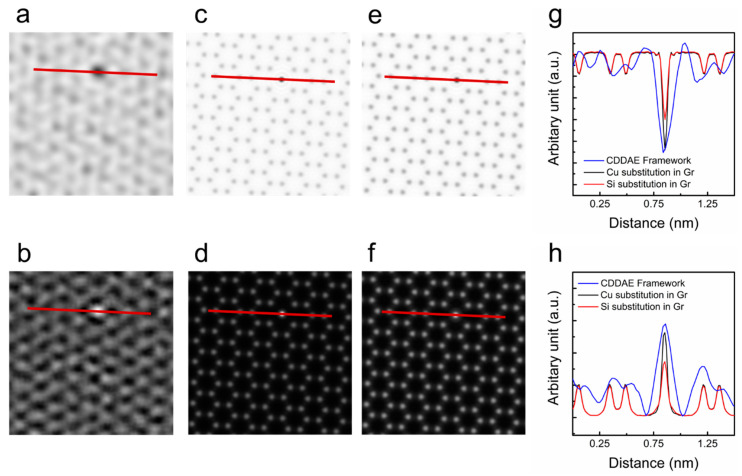
(**a**) Phase image and (**b**) amplitude image of the reconstructed exit wave, phase, and amplitude, from the atomic-resolution transmission electron microscopy (ARTEM) image by the CDDAE framework. Simulated (**c**) phase image and (**d**) amplitude image of the substituted Cu single atom in the graphene lattice. Simulated (**e**) phase image and (**f**) amplitude image of the substituted Si single atom in the graphene lattice. (**g**,**h**) The intensity profiles are plotted as solid red lines from left to right in each image.

The CDDAE framework achieved an accurate identification of substitutional atoms in graphene. The incorporation of substitutional atoms (Cr, Ti, Pd, Ni, Al, Cu, Si, B, or N) in the graphene lattice results in the etching and doping of graphene [27,28,29,30]. Such doping and etching by impurity atoms is useful in building novel nanostructures. TEMs have been used to analyze the impurity effects of graphene because TEM can identify impurity atoms and reconstructed novel nanostructures simultaneously. However, the identification of impurity atoms by TEM is difficult. In particular, substituted Cu and Si atoms are not discernible from TEM images, only because of misinterpretable atomic intensity differences [28]. Another obstacle is that the life-time of substitutional atoms is just a few seconds [28], which makes them difficult to observe in TEM. To distinguish Cu and Si atoms accurately, exit-wave reconstruction is necessary. In Figure 3, the exit-wave reconstructions performed by the CDDAE framework and MacTempas were compared. Figure 3c–f shows the phase and amplitude images simulated with MacTempas for both Cu and Si substitution in the graphene lattice. The reconstructed amplitude and phase with our experimental TEM condition made it possible to discern Cu and Si atoms with large differences between the amplitude and phase, as shown in Figure 3g,h. Comparing the phase and amplitude images of the exit-wave reconstruction by the CDDAE framework to those of the MacTempas results, we conclude that the substitutional atom in the graphene lattice is Cu. It is worth noting that the CDDAE framework can achieve exit-wave reconstruction with just one TEM image.

### 4.3. Denoising Performance Metrics

**Figure 4:**
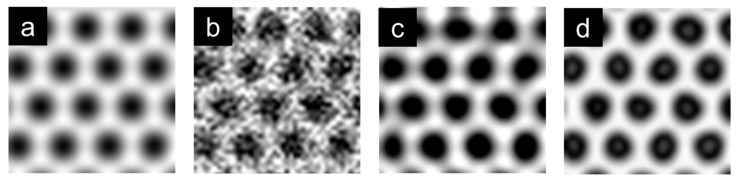
(**a**) Image simulation result of monolayer graphene using MacTempas; (**b**) Noise-added simulation image; (**c**) Denoising result of the noise-added image with the Wiener filter; (**d**) Denoising result of the noise-added image with the CDDAE framework.

*I*_r_, the result of image simulation through CTF and the reconstructed exit wave in the CDDAE framework, is a nonlinear denoising solution of the input image. A conventional denoising method, the Wiener filter provides a linear denoising solution [14]. To compare the performance of the two methods mentioned earlier, an image simulation dataset of monolayer graphene has been built using MacTempas, and detector noise has been added, so that of the SNR is 9.1164 according to the actual TEM conditions, as shown in Figure 4a,b.

The results of denoising the noise-added dataset with the Wiener filter and CDDAE framework are shown in Figure 4c,d. The quality of the images was evaluated by calculating SNR, PSNR, and SSIM. The average values of SNRs, PSNRs, and SSIMs of the images are summarized in Table 2. After filtering with the Wiener filter and the CDDAE framework, SNR, PSNR, and SSIM of each image were improved. Comparing the results, the CDDAE framework performed better for all three values. These results indicate that performance can be improved by the nonlinear denoising solution. Furthermore, it is evident that the reconstructed exit wave from the CDDAE framework is the CTF deconvolution solution.

## 5. Conclusions

In this study, we successfully demonstrate a deep learning framework, the CDDAE framework, for denoising and reconstructing the exit wave of ARTEM images. The CDDAE framework obtains the exit wave from a separated decoder, which decodes the phase and amplitude from the original images. The framework we proposed aimed to achieve the development of the conventional method, the use of a precalculated CTF, by finding a nonlinear solution through backpropagation.

The output of the image simulation in the CDDAE framework is a nonlinear denoising solution for ARTEM images, which exceeds the performance of a conventional linear denoising solution.

The proposed framework was validated by reconstructing the exit wave of the ARTEM images of CVD-grown monolayer graphene and comparing it with the results of the conventional exit wave simulation. Based on our proposed framework, we were able to differentiate between substituted Cu single atoms in the graphene lattice and substituted Si single atoms using, for the first time, only one ARTEM image. In addition, we proved that our method could perform the denoising and reconstructing of the exit wave of a single-defocused image of 2D materials without a through focal series of images. By adopting the proposed method, the misinterpretation of the image can be reduced, and the accurate atomic information can be used for the structural and dynamics studies of 2D materials with ARTEM. We believe this pioneering work opens up new possibilities for the use of deep learning techniques in the field of atomic-scale 2D material research.

## Figures and Tables

**Table 1:** Detailed information on the convolutional dual-decoder autoencoder (CDDAE) network.

Encoder	Act	Output Shape	Parameters
Input image	-	1 × 128 × 128	-
Convolution 3 × 3	ReLU	64 × 128 × 128	1048576
Convolution 3 × 3	ReLU	64 × 128 × 128	1048576
Downsample		64 × 64 × 64	
Convolution 3 × 3	ReLU	128 × 64 × 64	524288
Convolution 3 × 3	ReLU	128 × 64 × 64	524288
Downsample		128 × 32 × 32	
**Decoder1**	**Act**	**Output Shape**	**Parameters**
Upsample		128 × 64 × 64	
Convolution 3 × 3	Tanh	128 × 64 × 64	524288
Convolution 3 × 3	Tanh	128 × 64 × 64	524288
Upsample		128 × 64 × 128	
Convolution 3 × 3	Tanh	64 × 128 × 128	1048576
Convolution 3 × 3	Tanh	64 × 128 × 128	1048576
Convolution 1 × 1		1 × 128 × 128	
**Decoder2**	**Act**	**Output Shape**	**Parameters**
Upsample		128 × 64 × 64	
Convolution 3 × 3	Tanh	128 × 64 × 64	524288
Convolution 3 × 3	Tanh	128 × 64 × 64	524288
Upsample		128 × 64 × 128	
Convolution 3 × 3	Tanh	64 × 128 × 128	1048576
Convolution 3 × 3	Tanh	64 × 128 × 128	1048576
Convolution 1 × 1		1 × 128 × 128	

**Table 2:** Average values of SNR, PSNR, SSIM for the Wiener-filtered dataset and CDDAE framework.

Method	SNR	PSNR	SSIM
Noise-added	9.1164	17.5501	0.5443
Wiener filtered	16.7889	25.2226	0.9229
CDDAE framework	18.3390	26.7727	0.9440

SNR—signal-to-noise ratio; PSNR—peak signal-to-noise ratio; SSIM—structural similarity index map.
